# Landscape drivers of genomic diversity and divergence in woodland Eucalyptus

**DOI:** 10.1111/mec.15287

**Published:** 2019-11-17

**Authors:** Kevin D Murray, Jasmine K Janes, Ashley Jones, Helen M Bothwell, Rose L Andrew, Justin O Borevitz

**Affiliations:** ^1^ Australian National University Canberra ACT Australia; ^2^ University of New England Armidale NSW Australia; ^3^ Vancouver Island University, Nanaimo BC Canada

**Keywords:** adaptation, angiosperms, ecological genetics, landscape genetics, population genetics ‐ empirical, speciation

## Abstract

Spatial genetic patterns are influenced by numerous factors, and they can vary even among coexisting, closely related species due to differences in dispersal and selection. *Eucalyptus* (L'Héritier 1789; the “eucalypts”) are foundation tree species that provide essential habitat and modulate ecosystem services throughout Australia. Here we present a study of landscape genomic variation in two woodland eucalypt species, using whole‐genome sequencing of 388 individuals of *Eucalyptus albens* and *Eucalyptus sideroxylon*. We found exceptionally high genetic diversity (*π* ≈ 0.05) and low genome‐wide, interspecific differentiation (*F*_ST_ = 0.15) and intraspecific differentiation between localities (*F*_ST_ ≈ 0.01–0.02). We found no support for strong, discrete population structure, but found substantial support for isolation by geographic distance (IBD) in both species. Using generalized dissimilarity modelling, we identified additional isolation by environment (IBE). *Eucalyptus albens* showed moderate IBD, and environmental variables have a small but significant amount of additional predictive power (i.e. IBE). *Eucalyptus sideroxylon* showed much stronger IBD and moderate IBE. These results highlight the vast adaptive potential of these species and set the stage for testing evolutionary hypotheses of interspecific adaptive differentiation across environments.

## 1 INTRODUCTION

In wild species, and especially plants, genetic variation is inherently spatial: individuals occur at specific locations, and allele frequencies differ across the landscape as a result of variation in demographic history, patterns of gene flow and heterogeneous selection pressures. Landscape genomics is the study of the geographic distribution of alleles within a species and the underlying processes that shape gene flow. By interrogating spatial genetic patterns, we may examine the historical drivers of local genetic isolation and potential adaptation, and use this knowledge to better manage species under a changing environment (Hoffmann et al., 2015).

A multitude of processes may drive the spatial patterns of genetic diversity within and between species. Individuals may cluster into discrete genetic groups, with reduced gene flow between subpopulations relative to within. There are many potential causes of such discrete structure, for example geographic barriers to gene flow or flowering time divergence. Individuals may also exhibit patterns of continuous isolation by geographic distance (IBD; Wright, 1943) or isolation by environment (IBE; Wang & Bradburd, 2014). IBD is indicated by a positive correlation between increasing genetic dissimilarity and geographic distance, and is observed when individuals are more likely to reproduce with geographically proximate individuals. IBE is indicated by a correlation between genetic dissimilarity and environmental dissimilarity, while controlling for IBD. IBE can have many causes, for example environmental effects on phenology altering flowering time, or impeded dispersal between habitats due to maladaptation to local conditions. Any of these three patterns of genetic isolation over the landscape (discrete structure, IBD or IBE) may occur within a given species. Importantly, these patterns describe genome‐wide phenomena, and while they may be influenced or initially generated by selection on adaptive alleles, their detection is not evidence of local adaptation. While factors affecting dispersal, such as landscape resistance (Spear, Balkenhol, Fortin, Mcrae, & Scribner, 2010; Wang & Bradburd, 2014; Zeller, McGarigal, & Whiteley, 2012), may vary across the landscape, much can be learned by applying these global, homogeneous, dissimilarity‐based methods for studying IBD and IBE, particularly when integrated with tests of discrete genetic structure.

The processes that influence spatial autocorrelation of allele frequencies require sophisticated statistical methods to disentangle. Continuous isolation by distance can lead to support for discrete population structure in analysis with genetic clustering methods like structure (Pritchard, Stephens, & Donnelly, 2000) and admixture (Frantz, Cellina, Krier, Schley, & Burke, 2009). However, recent methodological developments now allow joint estimation of IBD and discrete structure (conStruct; Bradburd, Coop, & Ralph, 2017). Spatial autocorrelation of environmental variables makes disentangling their effects from IBD challenging, and older methods like partial Mantel tests are beset with several flaws (e.g. assumption of linearity, high type I error rate; Guillot & Rousset, 2013). Generalized dissimilarity modelling (GDM; Ferrier, Drielsma, Manion, & Watson, 2002; Ferrier, Manion, Elith, & Richardson, 2007) is a method which can accurately discriminate the geographic and environmental contributions to genetic differentiation, even where effects are nonlinear. Equally important is the selection of variables appropriate to one's study system: Williams, Belbin, Austin, Stein, and Ferrier (2012) propose a comprehensive variable set and variable selection methodology specifically for ecological models of habitats. However sophisticated the methods used to detect isolation by environment, it is a pattern affecting the genomic background. Locally adaptive loci should stand out above this background and could be identified subsequently via a genome scan.

Genus *Eucalyptus* (L'Héritier; the “eucalypts”) is a speciose lineage of trees and large shrubs that includes the keystone species of many Australian habitats. Box‐gum grassy woodlands are one such habitat, and while once common in south‐eastern Australia, their conversion to agricultural land has reduced their range significantly (NSW Scientific Committee, 2002). We sought to examine spatial genetic patterns in two foundation species of these grassy woodlands, *Eucalyptus albens* (Benth.; “white box”) and *Eucalyptus sideroxylon* (A. Cunn. ex Wools; “mugga ironbark”). The prevalence of discrete population structure, IBD and/or IBE has been studied in several eucalypt species (e.g. Andrew, Peakall, Wallis, & Foley, 2007; Andrew et al., 2005; Jones, Vaillancourt, & Potts, 2007; Jordan, Hoffmann, Dillon, & Prober, 2017; Rutherford et al., 2018; Steane, Conod, Jones, Vaillancourt, & Potts, 2006; Steane et al., 2015; Steane et al., 2014; Supple et al., 2018). Although eucalypts have very limited seed dispersal, they generally preferentially outcross and are pollinated by generalist bird and insect pollinators, both of which contribute to their spatial genetic structure (Booth, 2017; Potts & Gore, 1995; Williams & Woinarski, 1997). Spatial genetic autocorrelation is strong within populations, but tends to be weak at larger scales; for example, isolation by distance between localities is only apparent between localities separated by more than 500 km in *E. melliodora* (Supple et al., 2018). While many studies have tested for and found discrete genetic structure (e.g. in *E. globulus*; Steane et al., 2006), strong discrete genetic structure uncorrelated with geography has been reported less commonly in widespread eucalypt species (e.g. in *E. salubris*; Steane et al., 2015). In any case, given the likely conflation of IBD and discrete population structure by traditional genetic clustering methods (Bradburd et al., 2017; Frantz et al., 2009), the relative extent of IBD and discrete structure remains an open question in many species. Correlation between genetic variation and environment has been observed in many forms, including IBE (e.g. Supple et al., 2018) and genotype‐environment associations (e.g. Dillon et al., 2014; Jordan et al., 2017; Steane, Mclean, et al., 2017a; Steane, Potts, et al., 2017b; Steane et al., 2014).

We aimed to determine the relative influence of the various factors contributing to landscape‐scale spatial genetic patterns in *E. albens* and *E. sideroxylon*. The large estimated census sizes (González‐Orozco et al., 2016) of both species led us to predict that these species would exhibit high genetic diversity. The reproductive ecology and extensive latitudinal geographic ranges of these species, and previous results for closely related species, led us to expect weak patterns of IBD and little discrete population structure orthogonal to IBD in both these species. Given gene‐environment associations observed in closely related species, we also predicted that isolation by environment would be observed, particularly associations between genetic distance and variables describing the availability of and demand for moisture and nutrients. To test these hypotheses, we generated whole‐genome sequence data for 215 and 173 individuals of *E. albens* and *E. sideroxylon*, respectively. We quantified intraspecific genetic variation across the landscape, determined the extent of both continuous isolation by distance and isolation by environment and assessed discrete population structure independent of IBD.

## 2 METHODS

### 2.1 Study system

**Figure 1:**
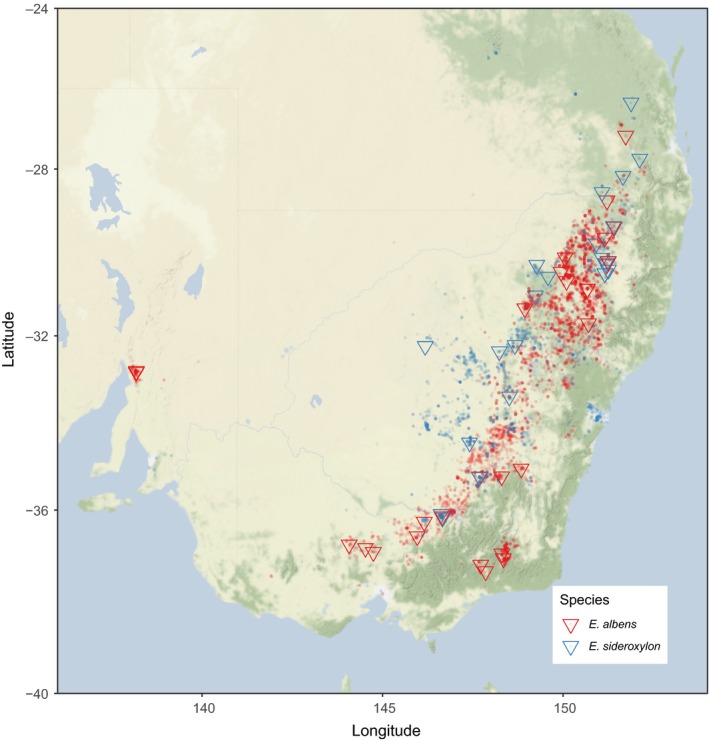
Focal species occurrence records and sampling localities. Geolocated occurrence records (±1 km accuracy) for Eucalyptus *albens* and *E. sideroxylon* obtained from the Atlas of Living Australia are overlain on a map of south‐eastern Australia. Sampling localities used in this study are indicated by large triangles [Colour figure can be viewed at http://wileyonlinelibrary.com]

The genus *Eucalyptus* (L'Héritier 1789; the “eucalypts”) is described as a highly speciose lineage of trees and large shrubs within family *Myrtaceae*. Of the more than 800 described species (Nicolle, 2018; Pryor & Johnson, 1971) that have evolved over the last 70 My (Thornhill, Ho, Külheim, & Crisp, 2015), nearly all are endemic to the Australian continent, with a small number of species occurring in Indonesia, the Philippines and New Guinea. Here we focus on two woodland eucalypt species. *Eucalyptus albens* and *E. sideroxylon* are from different series (*Buxeales* and *Melliodorae*, respectively) within *Eucalyptus* section *Adnataria*. They are morphologically distinct, differing in bark type (box vs. ironbark) and flower size and colour (*E. sideroxylon* larger, sometimes pink‐red pigmented; Brooker & Kleinig, 2006; Boland et al., 2006; Costermans, 1983). Both generally occur inland of the Great Dividing Range, with *E. sideroxylon*'s range extending further inland, while *E. albens* extends further south and has disjunct populations in south‐east Victoria and South Australia (see Figure 1). While both species have discontinuous distributions, partly as a result of post‐European land clearing, *E. sideroxylon*'s distribution is believed to have been more discontinuous precolonization (Costermans, 1983). Despite their largely sympatric distributions, there appears to be some niche specialization between these species, with *E. albens* occupying more fertile soils and *E. sideroxylon* preferring drier, well‐drained, more gravelly soils (Boland et al., 2006; Costermans, 1983; Harden, 2000). Despite their classification into different series, there is evidence of ongoing gene flow between these species, with reports of hybrid zones (Pryor, 1953), as is common in *Eucalyptus* generally, and especially in section Adnataria (Griffin, Burgess, & Wolf, 1988).

### 2.2 Data acquisition

Samples used in this study were collected from naturally occurring trees of the target species throughout south‐eastern Australia. Leaf tissue and fruit were collected from 3 to 15 trees from each location, across 39 distinct locations (Figure 1). Sample identifiers, GPS locations and additional metadata are presented online (https://doi.org/10.6084/m9.figshare.7583291.v1). Sampling was performed between 2015 and 2017. Leaves were dried on silica gel, and 20–30 3 mm leaf hole punches were taken for DNA extraction (Harris Uni‐Core WB100039). Hole punches were added to 1.1‐ml minitubes (Axygen Scientific) with a 3‐mm ball bearing, frozen under liquid nitrogen and ground for 2 min using a TissueLyser (Qiagen). DNA extraction was performed using a 96‐well column‐based kit, Invisorb DNA Plant HTS 96 Kit/ C 96 well purifications (Stratec Molecular 7037300400). The protocol was performed following the manufacturer's instructions, except for the lysis incubation, which was extended from 1 to 2 hr.

Multiplexed, short‐read, whole‐genome shotgun DNA sequencing libraries were generated using a cost‐optimized, transposase‐based protocol (Jones, Borevitz, & Warthmann, 2018). Briefly, fluorometric DNA quantification was performed using a Quant‐iT™ high sensitivity dsDNA assay kit (Molecular Probes™ Q33120). DNA was diluted to 2 ng/μl, quantified again and then diluted to 0.8 ng/μl, normalizing concentrations across all samples. Then, 3 μl of each sample (2.24 ng) was transferred to a new plate with a small quantity of a Nextera™ tagment DNA enzyme (Illumina catalogue #15027865) to add adapters (tagmentation). This reaction was optimized to be 1/25th of manufacturer's protocol, to save reagents and increase throughput. Custom index primers were used to amplify the libraries during 13 cycles of PCR (primer sequences provided in Jones et al., 2018). Libraries were purified and size‐selected with a combination of bead‐ and electrophoresis‐based methods, selecting fragments with insert sizes between 200 and 500 bp. These purified libraries were sequenced on a variety of Illumina platforms, with most libraries sequenced on multiple runs across both NextSeq 500 and NovoSeq 2000 instruments at the Biomolecular Resource Facility, ANU, and the Ramaciotti Center, UNSW. Multiple runs were pooled by sample to obtain sufficient coverage.

### 2.3 Alignment and polymorphism detection

**Figure 2:**
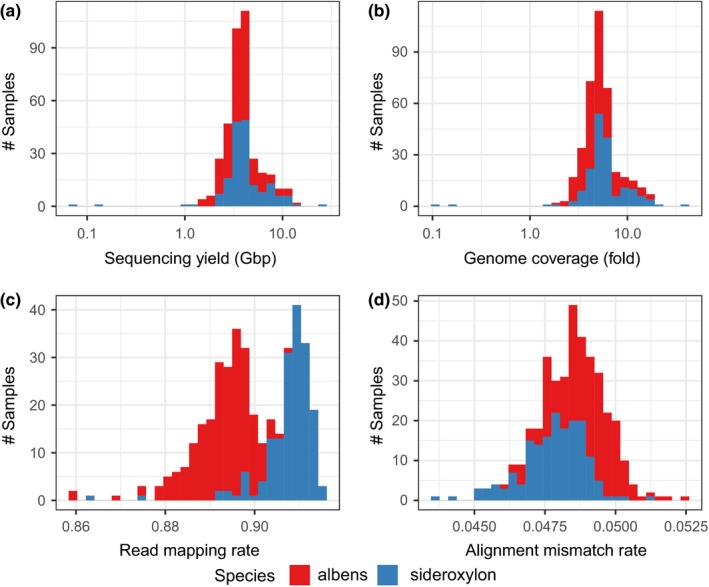
Whole‐genome sequencing yield and alignment statistics. (a) Post‐QC raw sequencing yield in bases, showing most samples yielded between 3 Gbp and 10 Gbp. (b) *Eucalyptus grandis* genome coverage (total sum of aligned bases). (c) Read alignment rate, (d), proportion of aligned bases which do not match the *E. grandis* reference genome. Overall, we have consistent, moderate coverage (median 5.2‐fold), although both read mapping and alignment mismatch rates suggest a reference bias between species (with *E. sideroxylon* appearing less distant). NB: histogram bars are stacked [Colour figure can be viewed at http://wileyonlinelibrary.com]

Sequencing yielded between 3 Gbp and 10 Gbp per sample, pooled across all sequencing runs (see Figure 2). Raw sequence data were quality filtered using adapterremoval (Schubert, Lindgreen, & Orlando, 2016), removing adaptor sequences, trimming low‐quality (<Q25) subsequences and merging overlapping read pairs. We used bwa mem version 0.7.15 (Li, 2013; Li & Durbin, 2009) to align short reads using default alignment parameters to the *Eucalyptus grandis* reference genome (genome size 640 Mbp), with an assembled *E. grandis* chloroplast added to the nuclear genome assembly (HM347959; Paiva et al., 2011; see Alwadani, Janes, & Andrew, 2019 for an analysis of chloroplast variation in this data set). Across all samples, 90% of reads were aligned to the *E. grandis* reference, with an average alignment mismatch rate of 4.8%. Both read mapping and alignment mismatch rates suggest a reference bias between species (with *E. sideroxylon* appearing less distant).

We detected short genomic variants using an efficient pipeline implementing the variant calling models contained in FreeBayes (Garrison & Marth, 2012) and bcftools mpileup (Li, 2011). As these tools are not internally parallelized, and the volume of data generated in this project was very large, we developed a genomic region‐parallelized system pipeline around these software. Briefly, this pipeline performs variant calling on each 100 kbp region of the *E. grandis* reference genome in parallel across hundreds of CPUs at once, before merging the candidate variants discovered in each region into a genome‐wide variant set. This variant set was then normalized with bcftools norm (Li, 2011), and block substitutions were decomposed to single nucleotide polymorphisms (SNPs) using vt decompose_blocksub (Tan, Abecasis, & Kang, 2015) and filtered with bcftools filter. We discarded variants with quality <10, fewer than five reads in total across all alleles in all samples and fewer than three reads supporting the alternate allele across all samples. In total, we discovered 132 million putative variants, of which 55 million were common (>10% minor allele frequency) SNPs within at least one species.

While many analyses require knowledge of exact genotypes for each sample, some methods (e.g. ANGSD; Korneliussen, Albrechtsen, & Nielsen, 2014) are able to represent uncertainty in individual genotypes through subsequent analyses. Given our low sequencing coverage, individual genotypes may have higher error than we desire, particularly in detecting heterozygosity. To address these concerns, we used ANGSD (Korneliussen et al., 2014) to detect putative variants and to calculate genotype likelihoods at each variable site. ANGSD considered loci only if there were >10 reads at a SNP (summed across at least 10 samples with data), considered reads only if they had a mapping quality >30, considered bases within reads only if they had a base quality score >20 and removed variants with a minor allele frequency <2%, with fewer than three reads supporting the alternate allele, or if the p‐value of the likelihood‐ratio test of nonzero minor allele frequency (i.e. test of polymorphism) was >0.001. Indel and block‐substitution variation is not considered by ANGSD. We used a region‐parallel approach similar to that used in variant calling to accelerate this computation. In total, ANGSD detected 55 million polymorphisms (variants with ≥10% minor allele frequency) across our samples.

From ANGSD likelihoods, we calculated several population genetic statistics. A two‐dimensional site‐frequency spectrum (SFS) between all *E. albens* and *E. sideroxylon* was calculated with realSFS (Nielsen, Korneliussen, Albrechtsen, Li, & Wang, 2012), then estimated genome‐wide *F*_ST_ between *E. albens* and *E. sideroxylon* using this two‐dimensional SFS as a prior (see Figure S10; ANGSD/realSFS estimates *F*_ST_ using the WC84 estimator). Using ngsdist (Fumagalli, Vieira, Linderoth, & Nielsen, 2014), we calculated intersample genetic distances for all samples that clustered into the two main species groups (based on kWIP distances). We estimated intersample covariance using pcangsd (Meisner & Albrechtsen, 2018). We calculated Euclidean distances from pcangsd covariances using the Gower transformation (*D_ij_* = *C_ii_* + *C_jj_* − 2*C_ij_*; Gower, 1985).

We implemented all steps in the above pipeline as a generic, modular workflow using the snakemake workflow manager (Köster & Rahmann, 2012). This snakemake pipeline allows parallelization of variant calling across genomic regions in a way that is abstracted from the execution environment. Project and cluster‐specific configuration of this pipeline are separate to pipeline code, allowing easy adaptation to other systems and data sets. This pipeline and associated scripts are open source and available online at https://github.com/kdmurray91/euc-dp14-workspace.

### 2.4 Population genetic analysis

We performed kmer‐based exploratory genetic analysis, to confirm sample identities and guide subsequent analyses. Genetic distances were estimated using kWIP, a kmer‐based estimator of genetic distance (Murray, Webers, Ong, Borevitz, & Warthmann, 2017). We first counted 21‐mers in unaligned, quality trimmed sequencing reads, after pooling all reads for each sample into one file. We estimated intersample genetic distances using the weighted inner product metric implemented in kWIP. Distances were estimated on each data subset (both *E. albens* and *E. sideroxylon*, and *E. albens* and *E. sideroxylon* separately) to allow subset‐specific weighting. We visualized these exploratory analyses using both hierarchical custering (hclust) and classical multidimensional scaling (cmdscale) in R 3.4 (R Core Team, 2018). In addition to kmer‐based estimates of genetic distance, we visualized the sample covariance (or genomic relationship matrix) as estimated by pcangsd in a similar fashion and compared these results visually.

To examine within‐locality diversity, a variety of population diversity metrics were employed. We calculated Nei's sample‐size corrected gene diversity (or expected heterozygosity, $H_{e}=\frac{2N}{2N-1}\frac{1-p_{l}^{2}-q_{l}^{2}}{L}$; Nei & Roychoudhury, 1974), using per‐locality allele frequencies calculated from expected genotypes by pcangsd. We displayed these measures of intra‐ and interlocation genetic diversity by plotting location estimates on a map of south‐eastern Australia using ggmap and Stamen map layers (Kahle & Wickham, 2013).

Traditional model‐based genetic clustering methods like structure (Pritchard et al., 2000) and admixture (Alexander, Novembre, & Lange, 2009) were designed to detect discrete population structure; therefore, they may perform poorly for continuously distributed natural populations in which isolation by distance is the primary driver of genetic structure (Frantz et al., 2009). ConStruct addresses this limitation by jointly modelling the effects of both continuous isolation by distance and discrete population structure on intersample relationships (Bradburd et al., 2017). As we expected continuously distributed landscape features to contribute to intersample genetic distances, we used conStruct to simultaneously test for discrete and continuous population structure. We used per‐locality allele frequencies calculated from pcangsd expected genotypes. We tested two distinct models separately for *E. albens* and *E. sideroxylon*, using the cross‐validation approach implemented in conStruct: a model similar to that used by structure and faststructure, and one allowing for isolation by distance within genetic clusters (“layers”). Layer contributions were calculated for all cross‐validation runs. To test for recent admixture between *E. sideroxylon* and *E. albens*, we used conStruct directly on the estimated genotypes, again performing cross‐validation and calculating layer contributions. To confirm that our findings were not specific to the recently released conStruct method, we ran faststructure (Raj, Stephens, & Pritchard, 2014) models of population structure on mpileup‐called SNP data. First, we used plink version 1.9 (Chang et al., 2015) to select a random 1% of variants and then ran fastStructure's structure.py with *K* ∈ {1, 2, 3, 4, 5}. We then used choosek.py to determine the model complexity that best described population structure in our data set, and we present admixture proportions for all *K* values.

We estimated the distribution of genome‐wide linkage disequilibrium by calculating inter‐SNP correlations and modelling correlation decay as a function of chromosomal position. Using the boringld R package (https://github.com/kdmurray91/boringld), we first calculated pairwise *r* ^2^ among SNPs in 30 kbp genomic windows with an overlap of 10 kbp between adjacent windows from FreeBayes‐called variants. Then, we fitted analytical models of the decay of *r* ^2^ as a function of inter‐SNP base pair distance using formulae derived by Hill and Weir (1988) and then calculated base pair distance to half‐maximal *r* ^2^ for each window. We summarized per‐window estimates of half‐maximal *r* ^2^ across all genome windows.

### 2.5 Landscape genomic analyses

We used GDM to test for isolation by distance without assuming a linear relationship between geographic and genetic distance using the gdm R package (Manion et al., 2018). Using genetic distances derived from pcangsd covariance, we modelled genetic distance as a function of geographic distance within each species. We calculated geographic distances between samples with *earth.dist* from the fossil R package (Vavrek, 2011). Models were constructed using individual‐level genetic and geographic distances, using three I‐spline knots. Only distance pairs with a geographic distance greater than 10 km (i.e. interlocation pairs) were considered. For each model, we examined the robustness of spline fits using jackknifing with 100 replicates. For each jackknife replicate, we removed all samples from a random 10% of sampling locations and fitted the GDM models as before. To perform cross‐validation of each model, we randomly partitioned data into training and test subsets comprising 90% and 10% of sampling locations, respectively. To compute the cross‐validation accuracy of models, we fit a GDM model on the training subset's pairwise distances and then computed cross‐validation accuracy as the correlation between actual genetic distances of samples from the 10% test data partition, and the corresponding distances predicted using the training subset GDM model. Note that as we subset sampling locations, the training:test ratio of pairwise distances is 81:19% due to the pairwise nature of distance calculations.

To assess isolation by environment, we first selected potentially relevant environmental variables based on a general methodology described by Williams et al. (2012). Variable values were extracted using the Atlas of Living Australia's (ALA) Spatial Portal Atlas of Living Australia, 2018). To determine which variables to include in models of IBE, we first performed forward selection within each category: Water, Energy and Soil (see Table S1). We excluded terrain and geoscientific variables, as these processes vary over finer spatial scales than our aggregated sampling resolution. In each forward selection run, we started with a GDM model of genetic distance as a function of geographic distance and proceeded by adding the variable that, when included, increased the proportion of deviance explained by the model by the largest amount. We terminated this process when no variable could explain at least 1% of additional deviance. We then combined forward‐selected variables across all categories into a candidate GDM model. To assess how representative our sampling was of each species' range, we compared distributions of each environmental variable from our sampling locations to distributions for ALA observation records for each species.

To refine candidate GDM models, and assess the importance and significance of constituent variables, we performed backward selection using the *gdm.varimp* function in the gdm package (Ferrier et al., 2007; Manion et al., 2018), with 100 permutation replicates for each step. For both species, the inflection point in decreased model deviance explained resulted in five variables retained for the final model (Figure S11). We then assessed the consistency of spline fits using the jackknifing approach described above. These new functions for variable selection and cross‐validation are available as an R package (https://github.com/kdmurray91/gdmhelpers).

## 3 RESULTS

### 3.1 Population genetic variation

**Figure 3:**
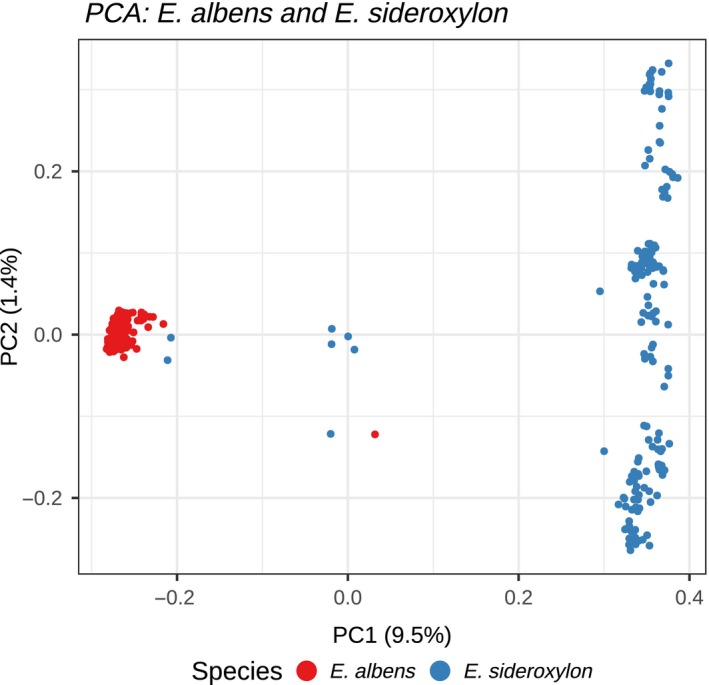
Principal component analysis (PCA) of genetic covariance of all samples. We plot the eigendecomposition of genomic covariance estimated from ANGSD genotype likelihoods using pcangsd. *Eucalyptus albens* and *E. sideroxylon* form discrete clusters separated on the first axis, which explains 9.5% of total genetic variance. Six samples fall between these clusters, likely representing recent admixture. These six samples include two individuals identified as putative hybrids in the field. We propose that *E. sideroxylon* is separated by PC2 as this species shows marginally stronger spatial genetic structure (see Figure 5); PC3 separates *E. albens* (not shown) [Colour figure can be viewed at http://wileyonlinelibrary.com]

After filtering unsupported or singleton variants, we discovered over 100 million candidate variants (varying slightly between software tools; Table S2). This equates to about 1/6th of all positions in the *Eucalyptus grandis* reference genome. Of these candidate variants, around 40% were not segregating (<10% minor allele frequency) in either *E. albens* and *E. sideroxylon*. Of the remaining approximately 60 million variants, over half were segregating in both species, with 22% private to *E. albens* and 23% private to *E. sideroxylon* (Table S2). ANGSD estimated interspecies genome‐wide *F*_ST_ between *E. albens* and *E. sideroxylon* to be 0.15; global intraspecific *F*_ST_ was 0.018 in *E. albens* and 0.017 in *E. sideroxylon*. Genomic PCA highlights this strong divergence and the presence of intermediate samples (putative hybrid individuals; see Figure 3).

*Eucalyptus albens* and *E. sideroxylon* had high genetic diversity. Expected heterozygosity within sampling locations ranged between 0.2 and 0.3 for both species, with *E. sideroxylon* having slightly lower mean location‐level diversity, particularly in northern localities. Both species exhibited high species‐wide genetic diversity (*E. sideroxylon H_e_* = 0.25, *π* = 0.053; *E. albens H_e_* = 0.26, *π* = 0.056). Background linkage disequilibrium (LD) decayed rapidly in both species (Figure S12). The median base pair distance to half‐maximal *r* ^2^ in *E. albens* was 92 bp (IQR 47–219 bp), while LD extended slightly further in *E. sideroxylon* (median 113 bp; IQR 55–264 bp).

### 3.2 Spatial genetic diversity and structure

**Figure 4:**
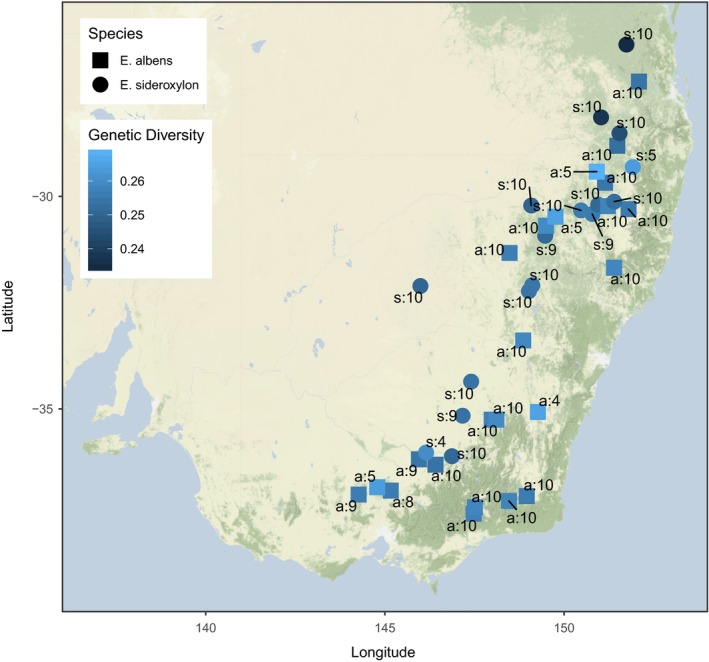
Geographic surface of genetic diversity superimposed on a map of south‐eastern Australia. Annotations describe species (s: or a: for *Eucalyptus sideroxylon* and *E. albens*, respectively) and the number of individuals per locality [Colour figure can be viewed at http://wileyonlinelibrary.com]

In general, genetic diversity was spread evenly over the range of our sampling in both species (Figure 4). Both *π* and *H_e_* are almost equal across all locations sampled in *E. albens*, while genetic diversity in *E. sideroxylon* declined very slightly in locations towards the north of our sampling.

### 3.3 No discrete but continuous population structure

**Figure 5:**
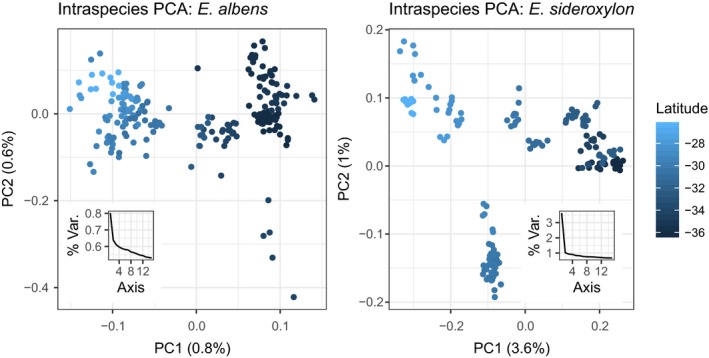
Principal component analysis (PCA) of *Eucalyptus albens* and *E. sideroxylon* individual genotypes. Axes describe eigendecomposition of pcangsd estimates of sample covariance. Individuals are coloured by latitude, the primary axis of variation in species' distributions. Insets show the distribution of leading eigenvalues. Note the absence of strong discrete clusters, the strong trend in PC1 across latitude and the low proportion of genetic variance explained by each leading axis. We plot PC1 across a map of the study area for both species in Figure S13 and equivalent biplots with PC1, PC2 and PC3 in Figure S14 [Colour figure can be viewed at http://wileyonlinelibrary.com]

Neither *E. albens* or *E. sideroxylon* exhibited strong signs of discrete population structure in a PCA of intrasample genetic covariance as estimated by pcangsd (Figure 5). Leading principal component axes explained little of the overall genomic variance between samples (0.8% and 0.6% in *E. albens*, 3.6% and 1.0% in *E. sideroxylon*). In each species, the leading principal component axis was correlated with latitude, suggesting isolation by geographic distance (*E. albens r* ^2^ = .92, *p* < .0001; *E. sideroxylon r* ^2^ = .87, *p* < .0001).

**Figure 6:**
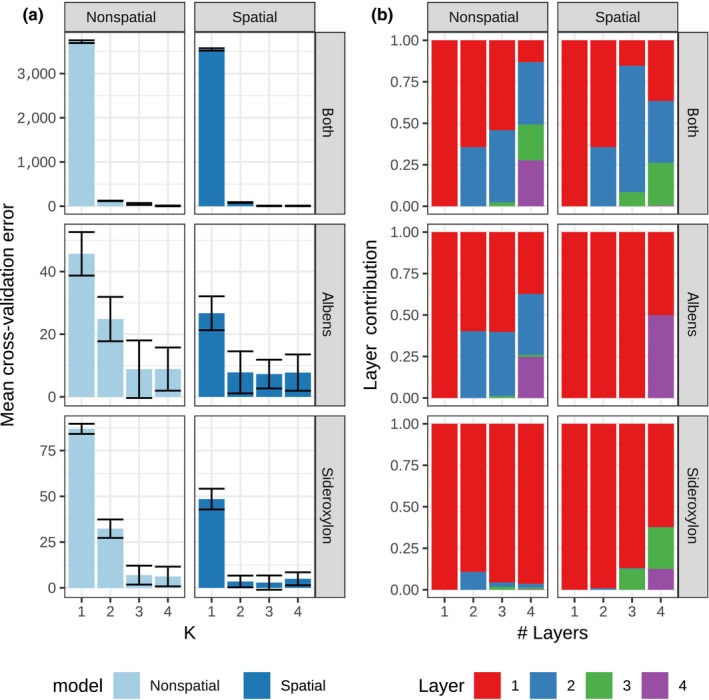
Cross‐validation of conStruct models of continuous and discrete population structure. (a) Model cross‐validation error, means ± *SD*. (b) Layer contribution to model explanatory power within each model with “# Layers.” Nonspatial: construct models that do not account for IBD, Spatial: construct models that allow for IBD within each population layer. Plots rows are for data sets with all localities across both *Eucalyptus albens* and *E. sideroxylon* (“both”) or within each species [Colour figure can be viewed at http://wileyonlinelibrary.com]

Joint estimation of continuous isolation by distance and discrete population structure indicated both species likely form single, continuous populations, with clinal structure influenced by strong IBD. When accounting for IBD in conStruct, cross‐validation of conStruct models suggested either one or two populations in both species (Figure 6). In models with two population layers, the second layer contributed very little additional predictive accuracy. The second layer in such models had no strong signal of IBD (Figure S15). This second layer could describe a small contribution of interspecies introgression to extant genetic diversity or could represent “homogeneous minimum layer membership,” an artefact produced by conStruct when there are significant levels of missing data (Bradburd et al., 2017). ConStruct models that did not allow continuous isolation by distance required at least two populations to achieve similar predictive accuracy (Figure 6). faststructure models fit to a subset of hard‐called SNPs confirmed these findings (Figure S16).

#### 3.3.1 Interspecific gene flow

We detected signals suggesting ongoing interspecies gene flow. Six samples were intermediate between *E. albens* and *E. sideroxylon*, being both intermediate in PCA (Figure 3), and having interspecies admixture proportions between 30% and 70% (Figure S17). Two of these samples were identified as putative hybrids in the field. Putative hybrids were found across three localities, and both *E. albens* and *E. sideroxlyon* were present at these localities. Mantel tests of interspecies distance pairs showed weak but statistically significant correlation between genetic distance and geographic distance, indicating that colocated *E. albens* and *E. sideroxylon* had lower genetic distance than geographically distant samples. This pattern could be due to interseries gene flow and is not predicted by incomplete lineage sorting, but could also be caused by certain demographic histories (e.g. expansion from shared ancestral refugia). Individual admixture proportions estimated by conStruct models supported the status of these six samples as recent hybrids (Figure S17). Additionally, conStruct models suggested a variable, small proportion (between 0% and 10%; Figure S17) of admixture from *E. albens* to *E. sideroxylon* (or vice versa). Additionally, more than half of all variants that were common in either species were common in both species (Table S2).

### 3.4 Isolation by distance and environment

**Figure 7:**
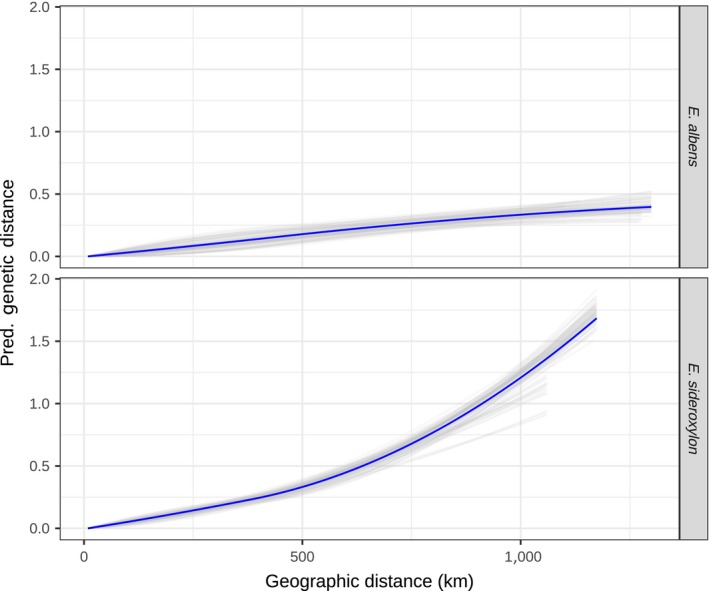
Geographic generalized dissimilarity modelling (GDM) shows strong isolation by distance. GDM model splines (blue) and jackknife replicate splines (grey) that best describe the association between geographic distance and genetic distance in each species. Geography‐only GDM models explain 26% of model deviance in *Eucalyptus albens* and 78% in *E. sideroxylon*. IBD appears to have an approximately linear trend in *E. albens*, while the strength of IBD increases for *E. sideroxylon* localities separated by more than 500 km [Colour figure can be viewed at http://wileyonlinelibrary.com]

Isolation by distance was moderately strong and largely linear in both species. Using GDM to model genetic distance as a function of geographic distance, we found *E. albens* to have moderately strong, almost linear IBD, with models explaining approximately 26% of overall deviance (*p* < .001; Figure 7). Meanwhile, *E. sideroxylon* exhibited very strong IBD, with models explaining 78% of overall deviance (*p* < .001; Figure 7). The relationships described by the best fit splines were robust to the removal of 10% of the sampling locations (i.e. jackknifing; Figure 7).

**Figure 8:**
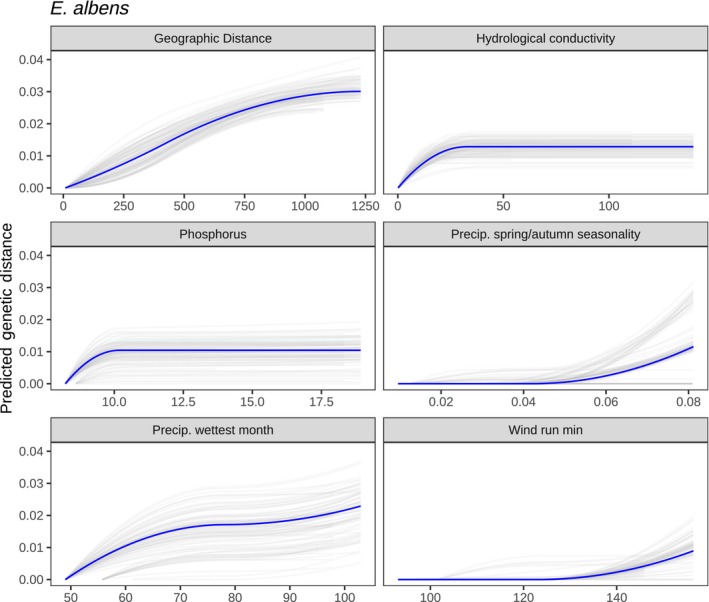
Generalized dissimilarity modelling (GDM) spline fits for *Eucalyptus albens*. To test the robustness of GDM predictive splines, models were rerun with 10% of sampling locations removed in each data set. Each panel showed the range of spline fits among the 100 jackknife replicates (grey) and the full data (blue) [Colour figure can be viewed at http://wileyonlinelibrary.com]

In the GDM analysis with environmental predictors, *E. albens* showed moderate isolation by environment, particularly driven by precipitation and substrate‐related environmental variables. Forward selection identified 11 candidate environmental covariates, each able to explain at least 1% additional deviance. Backward selection on these 11 variables identified substrate hydrological conductivity, substrate phosphorus concentration, spring/autumn precipitation seasonality, precipitation of the wettest quarter and total wind run as contributing the highest predictive power (Table S3). Overall, this model explained 31% of total deviance (*p* < .001), 7% higher than a model containing only geographic distance. Cross‐validation showed this model to have reasonable predictive accuracy; the correlation between predicted and true genetic distances was *r* ^2^ = .33, roughly equal to the percentage of deviance explained (Figures S18 and S19). For most variables, splines of best fit were robust to removal of 10% of sampling locations, although some variables had high uncertainty (e.g. precipitation of the wettest month), and other variables showed bimodal distributions of spline fits (e.g. autumn/spring precipitation seasonality; Figure 8).

**Figure 9:**
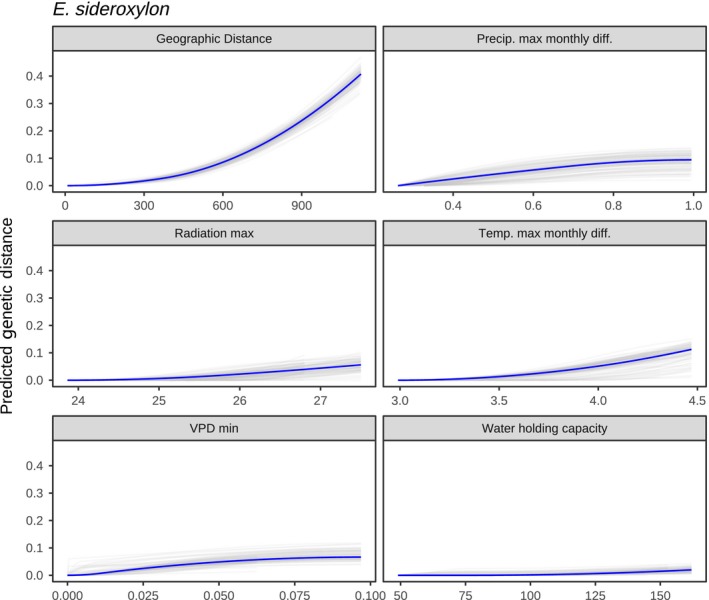
Generalized dissimilarity modelling (GDM) spline fits for *Eucalyptus sideroxylon*. To test the robustness of GDM predictive splines, models were rerun with 10% of sampling locations removed in each data set. Each panel showed the range of spline fits among the 100 jackknife replicates (grey) and the full data (blue) [Colour figure can be viewed at http://wileyonlinelibrary.com]

Similarly, *E. sideroxylon* showed somewhat stronger isolation by environment than *E. albens*, primarily driven by environmental variables describing the timing, availability and demand for moisture. Forward selection identified 12 candidate covariates, and backward selection identified maximum cloud‐adjusted solar radiation, maximum month‐on‐month differences in temperature and precipitation, maximal vapour pressure deficit and substrate water holding capacity as the five variables with highest predictive power (Table S3). Again, the overall model was highly significant (*p* < .001), explained 90% of total deviance (12% higher than a model containing only geographic distance) and had very high mean cross‐validation predictive accuracy (*r* ^2^ = .90; Figures S18 and S19). Splines of best fit were robust to removal of 10% of sampling locations for all predictors, with low uncertainty in spline fits across jackknifing replicates Figure 9.

## 4 DISCUSSION

### 4.1 Genetic diversity

Common, widespread eucalypts generally exhibit large, continuous populations with high genetic diversity and low population divergence. We confirm this result with one of the first whole‐genome population resequencing studies in wild eucalypts (Kainer, Stone, Padovan, Foley, & Külheim, 2018; Silva‐Junior & Grattapaglia, 2015). We estimated intraspecies *F*_ST_ to be 0.017–0.018, lower than estimates from previous studies in a variety of eucalypt species (*Eucalyptus melliodora*: *F*_ST_ = 0.04, Supple et al., 2018; *E. globulus*: *F*_ST_ = 0.08, Jones, Steane, Potts, & Vaillancourt, 2002), although similar to estimates in other eucalypt species (*E. obliqua*: *F*_ST_ = 0.015, Bloomfield, Nevill, Potts, Vaillancourt, & Steane, 2011). These previous estimates are of similar magnitude to widespread tree species in other biomes, for example Oaks, Poplar and Pine (*Quercus robur*: *F*_ST_ = 0.07, Vakkari, Blom, Rusanen, Raisio, & Toivonen, 2006; *Q. engelmannii*: *F*_ST_ = 0.04, Ortego, Riordan, Gugger, & Sork, 2012; *Populus tremuloides*: *F*_ST_ = 0.03, Wyman, Bruneau, & Tremblay, 2003; *Pinus taeda*: *F*_ST_ = 0.04, Eckert et al., 2010; *P. contorta*: *F*_ST_ = 0.02, Yang, Yeh, & Yanchuk, 1996). This very weak genetic structure likely results from a combination of very large, stable effective population sizes, widespread ranges and high outcrossing rates (Williams & Woinarski, 1997).

Genetic diversity both across all individuals and within localities is high in both species, albeit slightly lower in *E. sideroxylon* than *E. albens*. Direct comparison of heterozygosiy estimates is difficult, given the large effects of marker type and filtering on heterozyosity values. Previous work indicated especially high allozyme diversity in *E. albens* (Prober & Brown, 1994). Linkage disequilibrium reported here is less extensive than in some previous reports (Silva‐Junior & Grattapaglia, 2015) and is more similar to older estimates of LD decay from wild individuals of *E. grandis* (Grattapaglia & Kirst, 2008) and *E. globulus* (Thavamanikumar, McManus, Tibbits, & Bossinger, 2011).

A crucial caveat to these results is that we predominantly sampled from mature trees which likely predate the extensive land clearing and habitat fragmentation that accompanied European colonization of Australia. The applicability of these results and conclusions to future generations of these species is uncertain. Individuals from later generations show reduced but still high genetic and/or phenotypic diversity in recent studies of related *Eucalyptus* species (Broadhurst, 2013; Jordan, Dillon, Prober, & Hoffmann, 2016; Supple et al., 2018), although these studies examined planted individuals, either in provenance trials or revegetation efforts (Costa e Silva, Hardner, Tilyard, & Potts, 2011). Further research on the differences in genetic diversity between remnant stands and younger cohorts is warranted.

### 4.2 Continuous genetic divergence

We observed continuous differentiation across the landscape within both species, driven both by geography and environment. This matches findings in most previous studies of genomic variation in eucalypts (Jordan et al., 2017; Steane et al., 2015, 2014; Supple et al., 2018). However, unlike previous studies, we found no support for strong discrete genetic structure. As seen in simulated and empirical studies of continuously distributed species (Bradburd et al., 2017; Frantz et al., 2009), we found statistical support for discrete population structure only when IBD was not incorporated into models of population structure. This conflation of IBD and discrete structure cements the conclusion that accurate determination of population structure in widespread species should use methods that can jointly estimate isolation by distance and discrete population structure.

We found very strong isolation by distance, particularly in *E. sideroxylon*. This is much stronger than in previous studies on related species at similar spatial scales. For example, weak isolation by distance occurs among populations in *E. melliodora*, with little correlation of genetic and geographic distance between pairs separated by less than 500 km (Supple et al., 2018; but see Andrew et al., 2005), and relatively weak IBD has been found in *E. microcarpa* (Jordan et al., 2017). Weak IBD may have technical and/or biological causes. Noisy reduced‐representation sequencing methods that have large error in estimating sample genotypes (e.g. in *E. melliodora*; Supple et al., 2018), and therefore genetic distances, may have led to underestimation of the correlation between genetic and geographic distances. The difference in resolution in the present study may be partly due to our use of pcangsd to calculate genetic distances, as it is designed to reduce the stochastic effects of low‐coverage sequencing on interindividual distances. Shirk, Landguth, and Cushman (2017) find distances based on PCA axes most accurately detect isolation by distance and environment, and pcangsd is analogous to PCA‐based distances in this context.

Strong IBD is likely a result of patterns of migration imposed by the reproductive ecology of eucalypts (Williams & Woinarski, 1997). Seed dispersal is limited in eucalypts, with pollen exchange accounting for the vast majority of migration among localities (Booth, 2017; Potts & Gore, 1995; Williams & Woinarski, 1997); recent analysis of chloroplast markers in box‐ironbark eucalypts supports this (Alwadani et al., 2019). Pollination is facilitated by generalist insect, bird and mammal pollinators in nearly all species (Potts & Gore, 1995; Williams & Woinarski, 1997). Most exchanges of pollen occur within a limited local range; however, migration events occur over much longer ranges with lower frequency (Williams & Woinarski, 1997). As a result, genes are readily exchanged far beyond immediate neighbours. We found the strength of IBD to be strikingly different between *E. sideroxylon* and *E. albens*. This finding suggests that, while pollen‐mediated gene flow is strong enough to limit discrete population structure in both species, gene flow at larger spatial scales is more restricted in *E. sideroxylon* than in *E. albens*. This goes against the expectation that the larger, more coloured flowers of *E. sideroxylon* attract more frequent bird pollination, leading to higher pollen motility. The allegedly less continuous historical distribution of *E. sideroxylon* (Brooker & Kleinig, 2006; Costermans, 1983) could have contributed to the stronger continuous structure observed in this species. These observations are also supported by lower local genetic diversity within *E. sideroxylon*, particularly in northern localities.

### 4.3 Isolation by environment

We observed isolation by environment in both species, primarily driven by variables describing the availability of water and nutrients to plants, with little influence of temperature. Permutation‐based variable testing showed only a small orthogonal contribution of environment to observed genetic distances, after accounting for geographic distance. Strong spatial autocorrelation of environment variables prevents fully disentangling geographic and environmental contributions to gene flow across the landscape. Exclusion of relevant environmental variables could cause underestimation of overall IBE, although the variable selection procedure employed here tested the contribution of a broad range of environmental variables concerning soil, geology, precipitation, temperature, wind, solar radiation and aridity. In most cases, inference of the environmental drivers of genomic differentiation appears robust to subsampling of localities. GDM models of isolation by distance and environment had high cross‐validation accuracy, and all were significant under locality‐wise permutation testing. While specific environmental variables selected as most important were not shared, the strength of IBE was similar in both species. Furthermore, the variables most predictive of genetic distance in both species described the availability and demand for moisture or soil fertility (nutrient or water availability). Despite local niche separation (Boland et al., 2006; Brooker & Kleinig, 2006; Costermans, 1983; Harden, 2000), the ranges of *E. albens* and *E. sideroxylon* overlap significantly (Figure 1) and therefore likely experience selection along similar macroscale clines (e.g. temperature, aridity).

Correlation of genetic and environmental variation is well established in Eucalyptus. Differences in climate and soil nitrogen can predict genetic differentiation in *E. melliodora* (Supple et al., 2018). Allele frequencies at certain SNPs were significantly correlated with aridity, temperature and rainfall in *E. tricarpa* (Steane et al., 2014), *E. loxophleba* (Steane, Mclean, et al., 2017a) and *E. microcarpa* (Jordan et al., 2017). Our use of environmental variables designed to interrogate the ecology of Australian plants (Williams et al., 2012) precludes direct comparison of IBE among studies at the level of specific variables. However, our results follow a similar general pattern to these previous studies of gene‐environment association in eucalypts.

### 4.4 Interspecific divergence and gene flow

About half of all common variants discovered in this study are common in both species, and we observed low genome‐wide divergence between *E. albens* and *E. sideroxylon* (*F*_ST_ = 0.15). Recent evidence suggests the genetic divergence is not strong at most genomic loci in many species, both in eucalypts (Rutherford et al., 2018) and more broadly (Andrew & Rieseberg, 2013; Wu, 2001). Additionally, low interspecific differentiation is expected theoretically given extremely large effective population sizes, long generation times and relatively recent radiation (González‐Orozco et al., 2016).

Interspecific gene flow between eucalypts has been observed many times, though probably occurs at a low rate in nature (Griffin et al., 1988). We made several observations suggestive of ongoing gene flow between *E. albens* and *E. sideroxylon* (Figures 3 and S17). We identified several putative hybrid individuals in the field and via PCA, and conStruct indicated a low but consistent proportion of interseries admixture. Hybridization between *E. albens* and *E. sideroxylon* has been demonstrated previously (Pryor, 1953), and more broadly, a systematic review by Griffin et al. (1988) showed species within Eucalyptus section Adnataria were found to hybridize at the highest rate of any section. The proportion of hybrids we observe here is of the same approximate magnitude as that observed in several other eucalypts in the subgenus *Symphomyrtus* (1%–3%; Williams & Woinarski, 1997). Hybridization between *E. albens* and *E. sideroxylon* occurs in spite of ecological differentiation, for example, in the form of limited local co‐occurrence, different tolerance of poor soils and aridity (Boland et al., 2006; Costermans, 1983; Harden, 2000) and relatively little overlap in flowering period (*E. albens*: January–June, *E. sideroxylon* May–November; Costermans, 1983; Brooker & Kleinig, 2006).

### 4.5 Conservation implications

To avoid extirpation, organisms must either adapt or migrate as environments change (Aitken, Yeaman, Holliday, Wang, & Curtis‐McLane, 2008). Our findings of high genetic diversity imply a large pool of variation accessible to natural selection. However, the long generation time of these trees makes it unlikely that natural selection on local standing variation alone can outpace anthropogenic changes in climate and land use; therefore, migration of better‐adapted alleles is required (Booth, 2017; Booth et al., 2015). While we show pollen must have been exchanged over relatively large distances at a rate historically sufficient to prevent strong differentiation between localities, natural rates of migration are unlikely to prevent range contractions (Aitken & Bemmels, 2016; Booth, 2017; Prober et al., 2015). Human assistance may be required to shift the ranges of these and many other woodland species (Butt, Pollock, & McAlpine, 2013; González‐Orozco et al., 2016; Supple et al., 2018).

Management interventions can take numerous forms. There is a temptation to use models of isolation by environment to guide selection of seed sources for assisted migration. However, we urge the utmost caution when doing so: these models of IBE are based on genome‐wide patterns among predominantly near‐neutral genetic variation and use predicted, interpolated environmental data. Such models could detect the historical influence of environment on genetic diversity, but there is no promise that these influences reflect what may happen in the future. We discourage the use of these results (or the results of any similar study) to severely narrow the range of seed sources used in revegetation to individuals from nearby the revegetated locality. Instead, these studies can suggest the geographic and environmental range over which climate‐adjusted provenancing can be conducted without introducing highly diverged germplasm. Studies of inbreeding in eucalypts find strong effects of selfing and local inbreeding (Hardner & Potts, 1995), but little outbreeding depression was observed beyond hundreds of metres among intraspecific crosses of Hardner, Potts, and Gore (1998). Outbreeding depression is observed in more distant crosses (e.g. by Larcombe, Costa e Silva J, Tilyard P, Gore P, & Potts BM., 2016; Lopez, Potts, & Tilyard, 2000). Such results reinforce the need for a restoration strategy that focuses on adaptive potential as much as pre‐adapted germplasm. Our advice matches that proposed in numerous recent syntheses of revegetation strategy (Broadhurst et al., 2008; Kardos & Shafer, 2018; Prober et al., 2015; Weeks et al., 2011), in particular “climate‐adjusted provenancing” (Prober et al., 2015). As an additional consideration, climate change is not the only anthropogenic risk to these species: the habitat these species inhabit has been cleared extensively since European colonization of Australia, with only a few per cent of the habitat remaining (NSW Scientific Committee, 2002). Perhaps the most effective management action would be the prevention of further deforestation and habitat fragmentation, both for these species and generally.

### 4.6 Future directions

All patterns reported here concern genome‐wide average effects; significant variation between loci in patterns described here likely exists. Investigating how variation in ancestry, population structure, interspecific differentiation and associations with environment differs across the genome requires whole‐genome data sets, and the data set and analysis pipeline we present here enable these analyses. In particular, our finding of low linkage disequilibrium implies that many reduced‐representation sequencing methods would provide data for just a fraction of all independent loci and therefore miss important segregating variation (Ahrens et al., 2018; Lowry et al., 2017).

Genotype‐environment association (GEA) studies could detect individual alleles which vary in frequency across some environmental cline, accounting for geography and genome‐wide patterns (as has been observed with reduced‐representation sequencing in related species, e.g. Steane, Mclean, et al., 2017a; Steane, Potts, et al., 2017b; Steane et al., 2014). Loci that have undergone selective sweeps could also be detected, shedding further light on recent evolution (Nielsen et al., 2005). Similarly, investigation of interspecies divergence at specific loci could highlight which loci are maintaining species boundaries in the face of gene flow (Strasburg et al., 2012). Finally, genome‐wide average ancestry may differ significantly from local ancestry at nearly all loci across the genome and could be examined in these species (e.g. using Local PCA; Li & Ralph, 2018).

## 5 CONCLUSIONS

In summary, we found high intraspecific genetic diversity, low genome‐wide divergence between *Eucalyptus albens* and *E. sideroxylon* and evidence of ongoing gene flow between these species. We found no evidence of strong, discrete population structure and uncovered strong continuous isolation by distance in both species. We also found that isolation by geographic distance accounts for most, but not all, of this continuous genetic structure, with environmental variables describing the availability and demand for moisture, temperature and substrate contributing to the pattern of IBE. Taken together, these results describe *E. albens* and *E. sideroxylon* as widespread species with high genetic diversity and strong isolation by distance. A small proportion of genetic variation is associated with climate; however, high levels of genetic diversity exist regionally and even within localities. This high genetic diversity implies these species have high adaptive potential, especially if enhanced by assisted migration. The crucial test of these species' survival will not be the level of understanding we gain about the intricacies of isolation by landscape, but rather the extent to which we utilize these and other species in large‐scale rehabilitation of degraded ecosystems.

## AUTHOR CONTRIBUTION

R.L.A., J.O.B., J.K.J., and K.D.M. designed this study; J.K.J., K.D.M., R.L.A., and A.J. created data used in this study; K.D.M. and R.L.A. designed and performed analyses presented in this study; K.D.M. wrote the first manuscript draft; All authors contributed to writing and review of the final manuscript.

## Data Availability

Raw sequencing data are available on the NCBI Sequence Read Archive, under project accession PRJNA578806 (Murray et al., 2019). Sample metadata and genome sequencing analysis code are available on GitHub at https://github.com/kdmurray91/euc-dp15-workspace. Supplementary metadata including sample identifiers, GPS locations and additional metadata are presented online (https://doi.org/10.6084/m9.figshare.7583291.v1).
