# Supplementary material for: Landscape drivers of genomic diversity and divergence in woodland Eucalyptus
Source: Mol Ecol. 2019 Nov 17;28(24):5232–47. doi: 10.1111/mec.15287 (PMC7065176; doi:10.1111/mec.15287)
Supplement: Supplementary file 1 [file MEC-28-5232-s001.pdf]

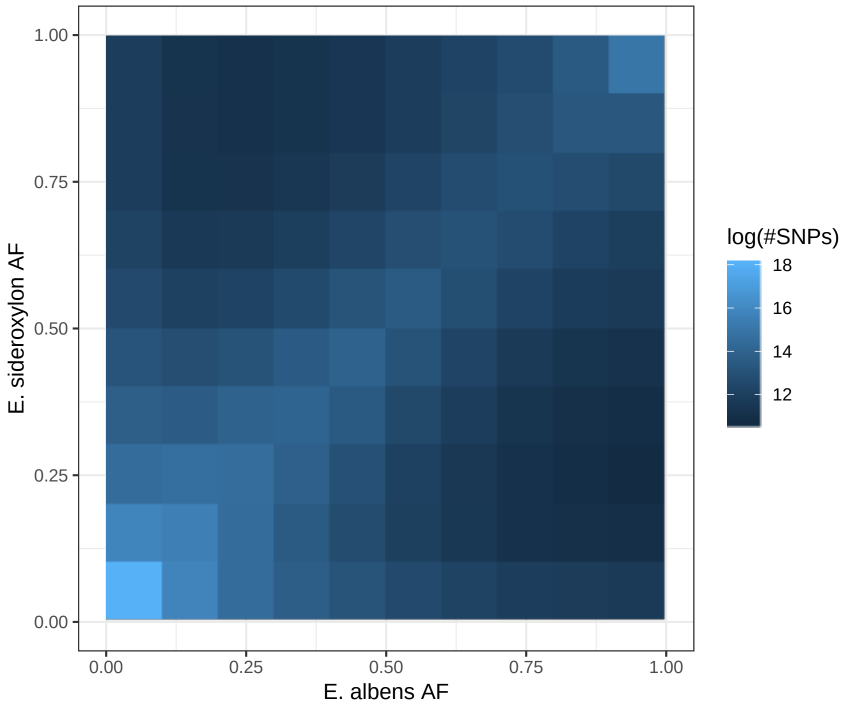

Figure 10: Two-dimensional site frequency spectrum between *E. albens* and *E. sideroxylon*.

Table 1: Environmental variables considered in forward selection of IBE models.

Abbreviated Name	Williams *et al.* Class	Name	Description
maxti	Energy	Temperature - coolest month max	Maximum temperature coolest month (°C)
maxtx	Energy	Temperature - month hottest maximum	Maximum temperature hottest month (°C)
minti	Energy	Temperature - coldest month min	Minimum temperature coldest month (°C)
mintx	Energy	Temperature - warmest month min	Minimum temperature warmest month (°C)
radni	Energy	Radiation - min month precipitation modified	Minimum month rainfall-modified solar radiation (MJ/m2/day)
radnx	Energy	Radiation - max month precipitation modified	Maximum month rainfall-modified solar radiation (MJ/m2/day)
rh2max	Energy	Humidity - month max relative	Maximum month relative humidity (%)
rh2min	Energy	Humidity - month min relative	Minimum month relative humidity (%)
rtimax	Energy	Temperature - max difference in min	Maximum difference in minimum temperatures (°C/day)
rtimin	Energy	Temperature - min difference in min	Minimum difference in minimum temperatures (°C/day)
rtxmax	Energy	Temperature - max difference in max	Maximum difference in maximum temperatures (°C/day)
rtxmin	Energy	Temperature - min difference in max	minimum difference in maximum temperatures (°C/day)
tmaxabsx	Energy	Temperature - max absolute mean max	Maximum month absolute mean maximum temperature (°C)
tminabsi	Energy	Temperature - min absolute mean min	Minimum month absolute mean minimum temperature (°C)
trngi	Energy	Temperature - min month diurnal range	Minimum month diurnal temperature range (°C)

Abbreviated Name	Williams *et al.* Class	Name	Description
trngx	Energy	Temperature - max month diurnal range	Maximum month diurnal temperature range (°C)
vpd2max	Energy	Vapour pressure deficit - month max	Maximum month vapour pressure deficit (KPa)
vpd2min	Energy	Vapour pressure deficit - month min	Minimum month vapour pressure deficit (KPa)
wind_windri	Energy	Wind run - month min	Wind run - month min (km/day)
wind_windrx	Energy	Wind run - month max	Wind run - month max (km/day)
wind_windspmax	Energy	Wind speed - month max 9am or 3pm	Wind speed - month max 9am or 3pm (m/s)
wind_windspmin	Energy	Wind speed - month min 9am or 3pm	Wind speed - month min 9am or 3pm (m/s)
substrate_bdensity	Soil	Bulk density	Solum average bulk density (Mg/m3)
substrate_calcrete	Soil	Calcrete	Calcrete in or below soil profile (presence)
substrate_clay	Soil	Clay %	Solum average median clay content (%)
substrate_coarse	Soil	Soils - coarse	Soils dominated by coarse fragments including ironstone (class)
substrate_ks_err	Soil	Hydrological conductivity - uncertainty	Solum average uncertainty of horizon saturated hydraulic conductivity estimates (index)
substrate_ksat	Soil	Hydrologic conductivity - average saturated	Solum average median horizon saturated hydraulic conductivity (mm/h)
substrate_nmnlconcn0	Soil	Nitrogen concentration pre-European	Pre-European estimate of mean annual concentration of mineral nitrogen in soil water (NMnlConc0.Base)
substrate_ntotn0	Soil	Nitrogen - plant-available pre-European	Pre-European estimate of mean annual store of total plant-available soil nitrogen (NTot0.Base)
substrate_nutrients	Soil	Nutrient status	Gross nutrient status (rating)

Abbreviated Name	Williams *et al.* Class	Name	Description
substrate_pmnlconcn0	Soil	Phosphorus pre-European	Pre-European estimate of mean annual concentration of dissolved phosphorus in soil water (PMnlConc0.Base)
substrate_pmnln0	Soil	Phosphorus - plant-available pre-European	Pre-European estimate of mean annual store of plant-available mineral phosphorus (PMnl0.Base)
substrate_soldepth	Soil	Soil depth	Solum depth (surface and subsoil layers) (metres)
substrate_solpawhc	Soil	Water holding capacity - plant-available	Plant-available soil water holding capacity (mm)
substrate_wr_unr	Soil	Unreliable water retention parameters	Solum average unreliable water retention parameters (index)
adefi	Water	Precipitation deficit - month max	Maximum month precipitation deficit (mm)
adefx	Water	Precipitation deficit - month min	Minimum month precipitation deficit (mm)
arid_max	Water	Aridity index - month max	Maximum month aridity index
arid_min	Water	Aridity index - month min	Minimum month aridity index
evapi	Water	Evaporation - month min	Minimum month evaporation (mm)
evapx	Water	Evaporation - month max	Maximum month evaporation (mm)
raini	Water	Precipitation - driest month	Precipitation of the driest month (mm)
rainx	Water	Precipitation - wettest month	Precipitation of the wettest month (mm)
rprecmax	Water	Precipitation - max difference between successive months	Greatest rainfall difference between successive months (mm/day)
rprecmin	Water	Precipitation - min difference between successive months	Least rainfall difference between successive months (mm/day)
slrain0	Water	Precipitation - annual (log) seasonality index	annual (log) rainfall seasonality index

Abbreviated Name	Williams *et al.* Class	Name	Description
slrain1	Water	Precipitation - summer or winter (log) season	summer or winter (log) rainfall season
slrain2	Water	Precipitation - spring or autumn (log) season	Spring or autumn (log) rainfall season
srain0mp	Water	Precipitation - annual seasonality ratio	annual rainfall seasonality ratio
srain1mp	Water	Precipitation - solstice seasonality ratio	Solstice rainfall seasonality ratio
srain2mp	Water	Precipitation - equinox seasonality ratio	Equinox rainfall seasonality ratio

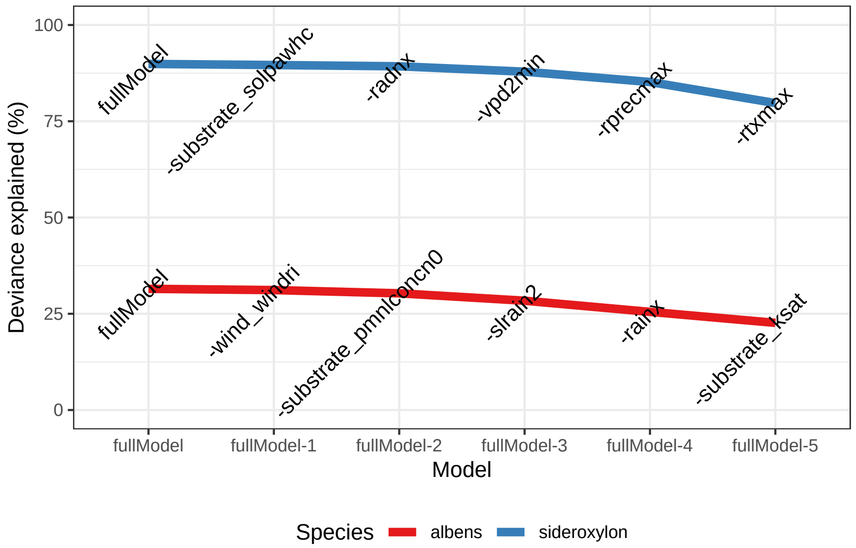

Figure 11: GDM model deviance explained during back-selection of variables. "fullModel" describes the model with all variables included. Each subsequent point removes one variable (per labels on plot). Please see Supplementary Table 1 for variable names

Table 2: SNP genotyping statistics.

Segregating in	Angsd SNPs
Neither species	78017065
Both species	29409038
E. albens	12407339
E. sideroxylon	12644030
Total	132477472

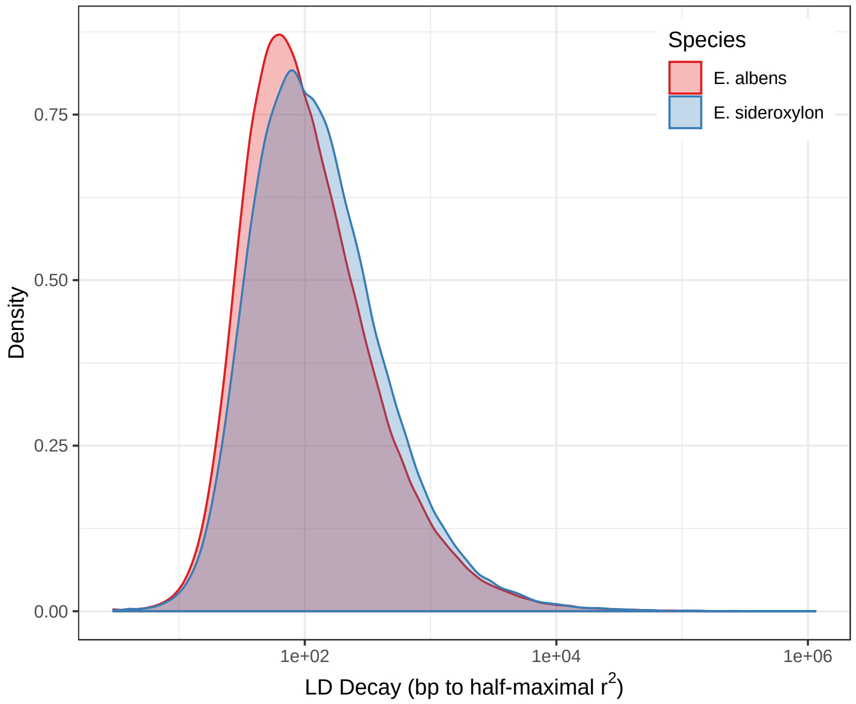

Figure 12: Distribution of LD extent for *E. albens* and *E. sideroxylon*. Here we show the distribution of LD extent, defined as the distance required for half-maximal decay in R^2, aggregated for all 1000000 bp genome windows

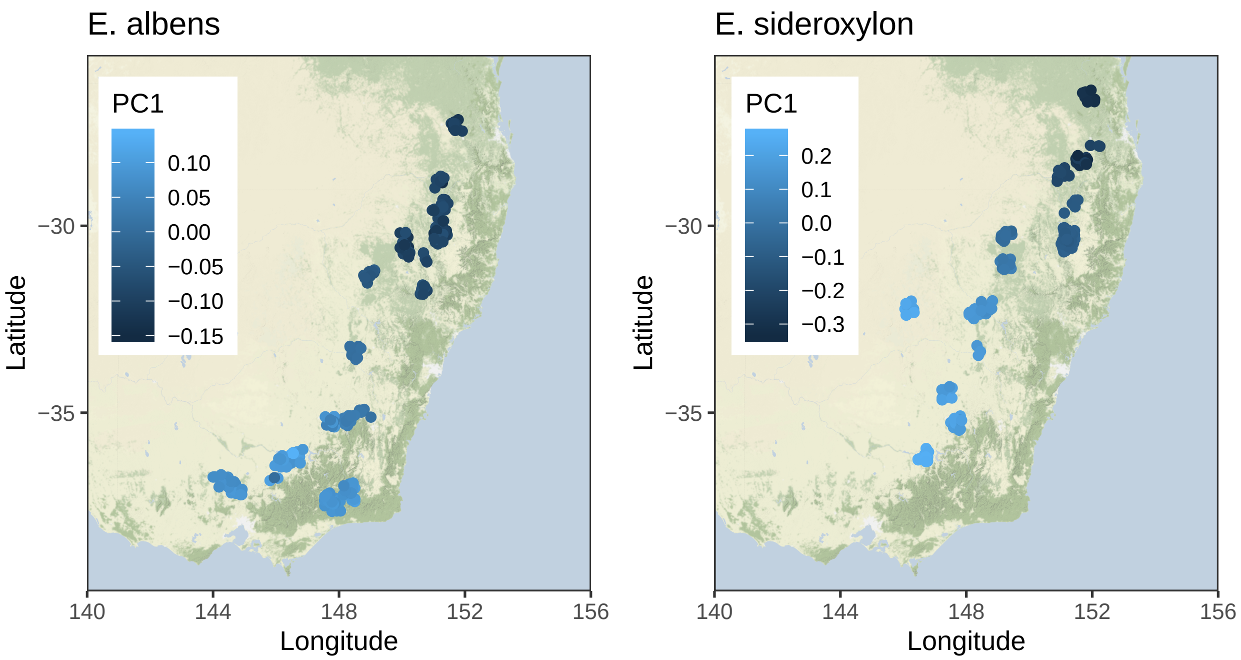

Figure 13: Geographic representation of PCA axes. Here we show the geographic patterns in PC axis 1 from each PCA in Figure 5. The first PCA axis explains 0.8% and 3.6% of total genetic variance in *E. albens* and *E. sideroxylon* respectively.

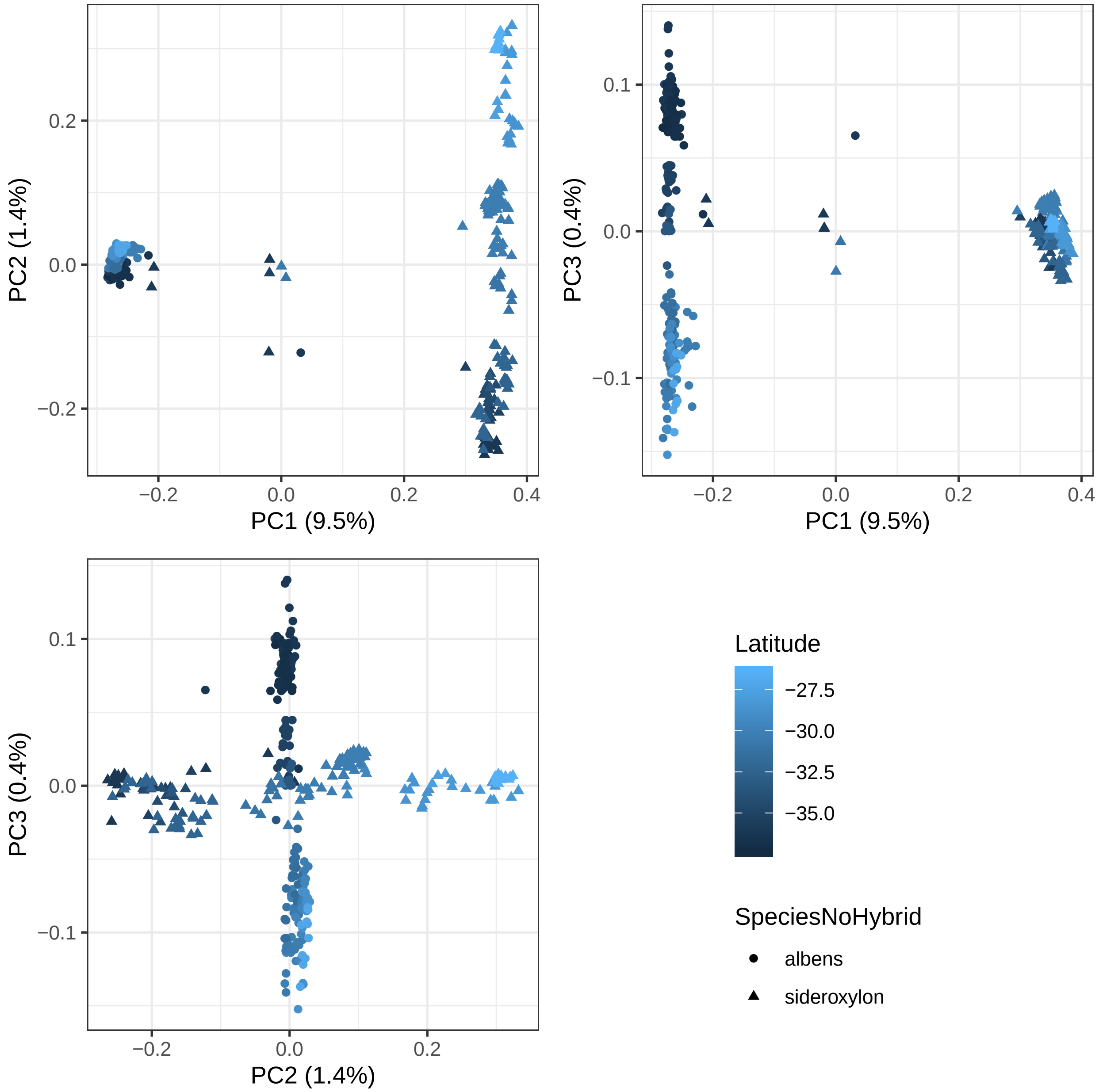

Figure 14: Higher-order PCA analysis of interspecific genetic distances. In Figure 3 we use PCA to display the first two axes of genomic variance across both *E. albens* and *E. sideroxlyon*. Here, we plot PC axis 3 in combination with PCA1 and 2, showing that PC1 separates indiviuals into the two species, PC2 separates *E. sideroxlyon* individuals, and PC3 separates *E. albens* individuals.

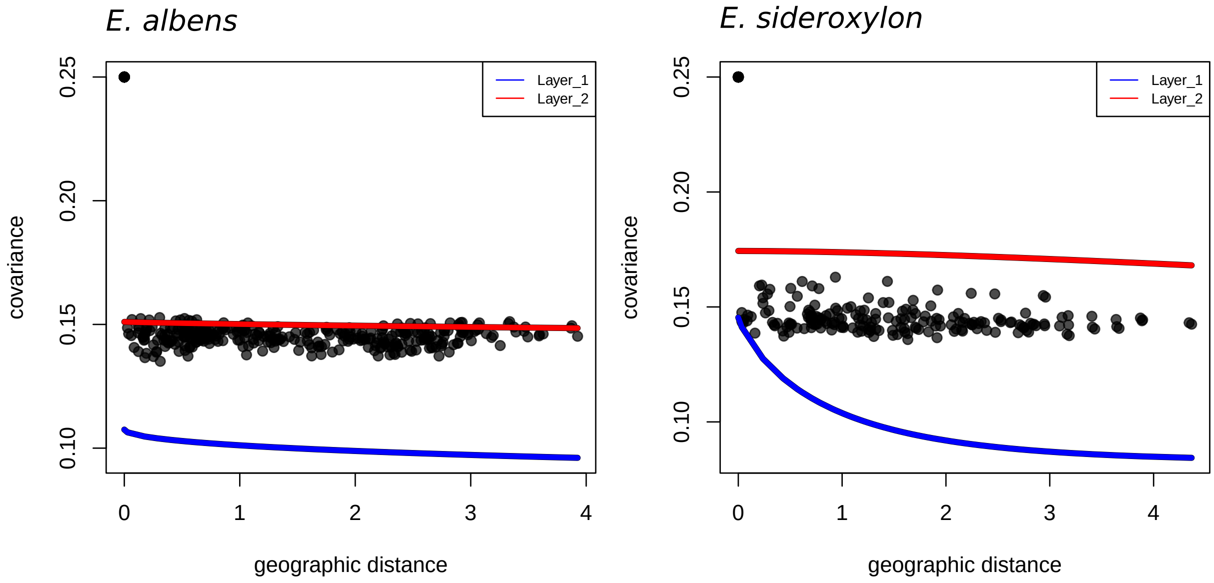

Figure 15: Decay of allelic covariance with distance in spatial conStruct models. ConStruct models isolation by distance as exponential decay of alleic covariance with geographic distance within each "layer" (i.e. subpopulation). Here, we present the modeled decay for each layer of construct models with two layers for each of *E. albens* and *E. sideroxylon*. While supported by cross-validation, the second layer in each model contributes very little to each models explanatory power, and has little evidence of IBD.

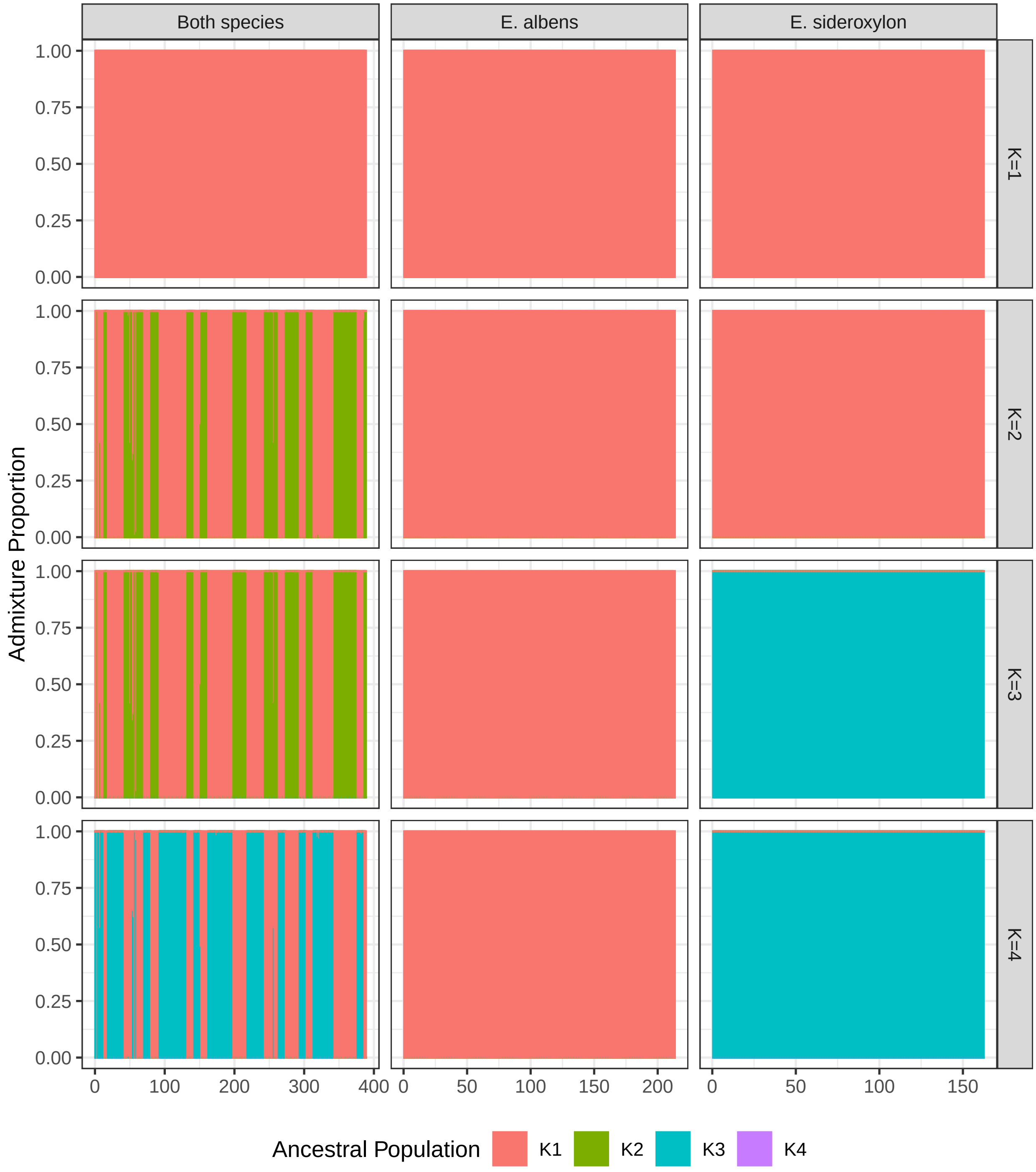

Figure 16: fastStructure analysis of population structure. FastStructure models of population structure were fit for $K \in \{1,2,3,4\}$ for all samples ("Both Sp."), and for non-outlier samples of each species ("E. albens"; "E. sideroxlyon" respectively). For each species, $K = 1$ maximised marginal likelihood and best explained structure, as determined by fastStructure's `chooseK.py`. For the whole dataset with both species, $K = 2$ maximised marginal likelihood.

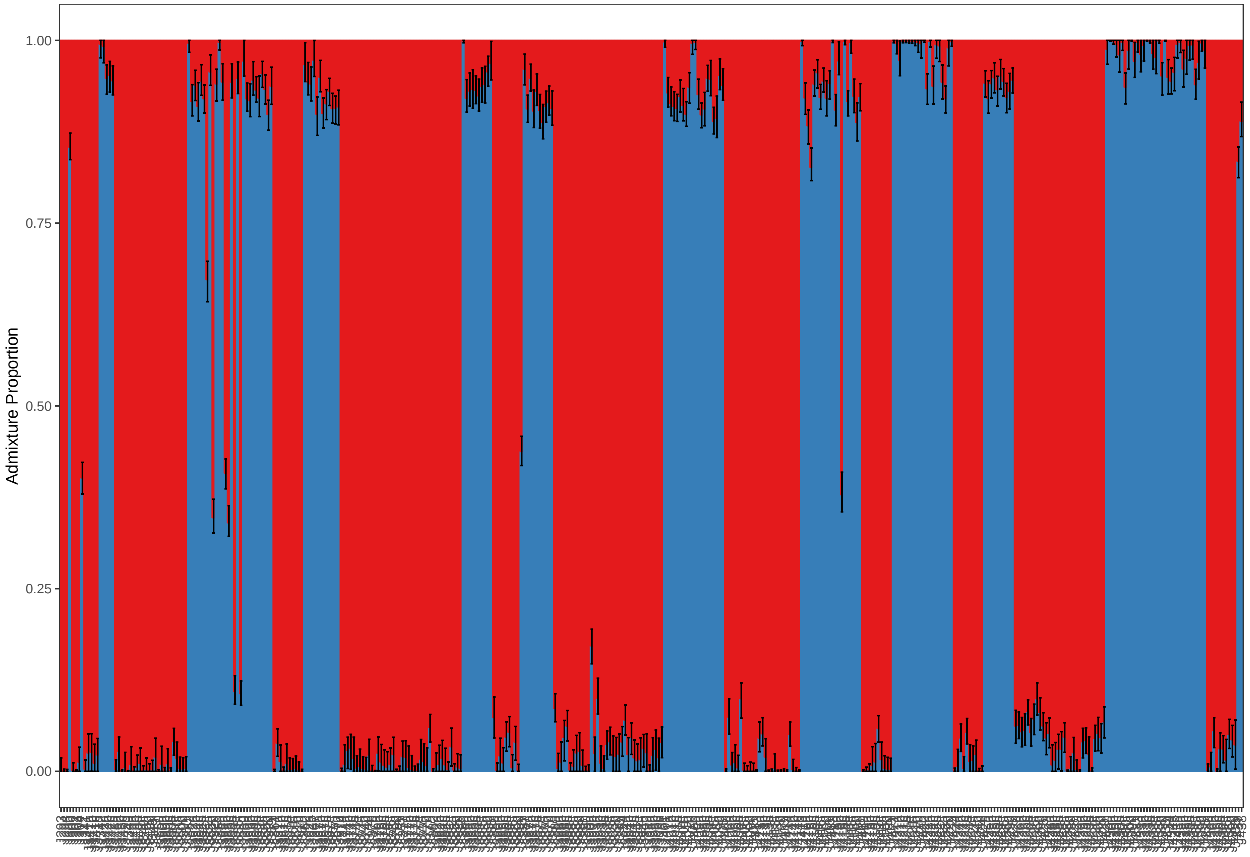

Figure 17: Individual-level conStruct analysis of all samples with two model layers. Admixture proportions are presented as means ± sd across 20 random subsets of 1 million SNPs. Note samples that appear as intermediates, suggesting they are recent interspecific hybrids.

Table 3: GDM model variables, model deviance explained, and variable-specific p-values.

E. albens		
Variable	% dev. expl.	Variable p-value
Geographic	31.475641	0
Hydrologic conductivity - average saturated	31.157494	0.05
Precipitation - wettest month	30.314927	0.02
Precipitation - spring or autumn (log) season	28.387513	0.13
Phosphorus pre-European	25.490866	0.19
Wind run - month min	22.645057	0.36
E. sideroxylon		
Variable	% dev. expl.	Variable p-value
Geographic	89.887027	0
Temperature - max difference in max	89.599473	0
Precipitation - max difference between successive months	89.265338	0
Vapour pressure deficit - month min	87.875363	0
Radiation - max month precipitation modified	85.197611	0.04
Water holding capacity - plant-available	79.720207	0.12

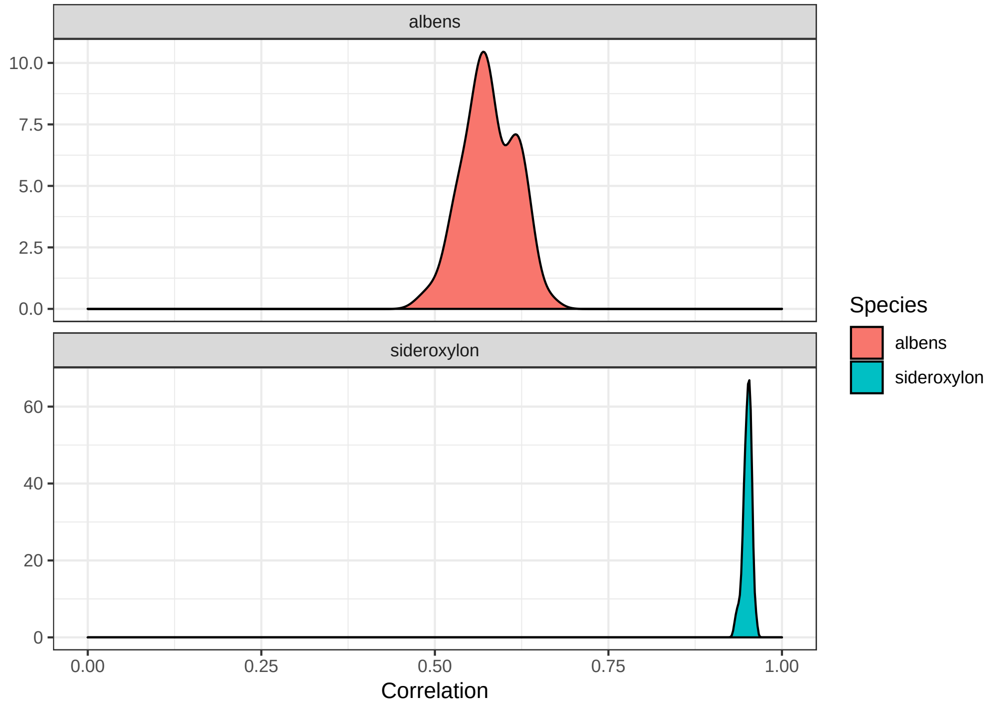

Figure 18: Cross-validation accuracy of best-fit GDM models for *E. albens* and *E. sideroxylon*. To test the predictive power of GDM models, GDM are fit on a training dataset with 10% of sampling locations removed in each dataset. The genetic distances of the remaining 10% of samples are predicted from their geographic and environmental data. Pearson's correlation is used to assess the goodness-of-fit between predicted and actual genetic distances.

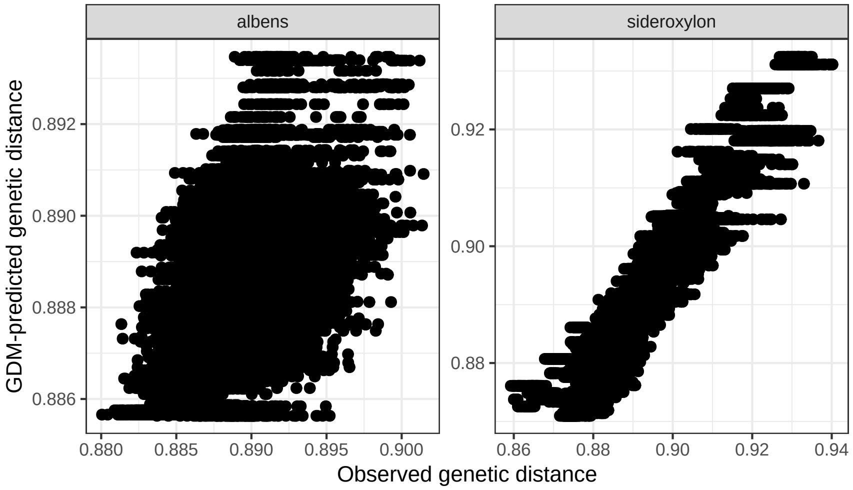

Figure 19: GDM model predictions vs observed genetic distances. GDM models predict genetic distance from geographic and environmental distances. Here, we plot the predicted genetic distances against observed genetic distances for each species' best GDM model (see Figures 8 and 9).
